# Estimating group differences in network models using moderation analysis

**DOI:** 10.3758/s13428-021-01637-y

**Published:** 2021-07-21

**Authors:** Jonas M. B. Haslbeck

**Affiliations:** Psychological Methods Group, University of Amsterdam, Amsterdam, Netherlands

**Keywords:** Network models, Group differences, Moderation

## Abstract

Statistical network models such as the Gaussian Graphical Model and the Ising model have become popular tools to analyze multivariate psychological datasets. In many applications, the goal is to compare such network models across groups. In this paper, I introduce a method to estimate group differences in network models that is based on moderation analysis. This method is attractive because it allows one to make comparisons across more than two groups for all parameters within a single model and because it is implemented for all commonly used cross-sectional network models. Next to introducing the method, I evaluate the performance of the proposed method and existing approaches in a simulation study. Finally, I provide a fully reproducible tutorial on how to use the proposed method to compare a network model across three groups using the R-package *mgm*.

## Introduction

Statistical network models such as the Gaussian Graphical Model (GGM), the Ising model or Mixed Graphical Models (MGMs) have become popular tools to analyze multivariate cross-sectional datasets (Epskamp et al., 2016; Epskamp et al., 2018; van Borkulo et al., 2014; Williams & Mulder, 2019; Haslbeck & Waldorp, 2020). In many of these applications one is interested in comparing such network models between two or more groups. For example, Fritz et al., (2018) compared the relations between resilience factors in a network model for adolescents who did or did not experience childhood adversity; van Loo et al., (2018) compared the relations between depression symptoms across various environmental and genetic risk factors; and Birkeland et al., (2091) investigated gender differences in the relations between PTSD symptoms after terror attacks.

There are already several methods available to estimate group differences in network models. The Network Comparison Test (NCT; van Borkulo et al.,, 2017), which is currently implemented for the GGM, the Ising model, and MGMs uses a permutation test to compare pairs of groups. The Fused Graphical Lasso (FGL; Danaher et al.,, 2014), which is currently available only for the GGM, compares two or more groups by applying a penalty term to group differences and performing model selection across different penalties. Epskamp et al., (2020) proposed a method that is based on iterative model search and pruning within the SEM framework. Finally, one can compare GGMs across two or more groups in a Bayesian framework by using a Bayes factor or thresholding the posterior of group differences (Williams et al., 2019).

In the present paper, I introduce a new method to estimate group differences in network models that is based on moderation analysis. Specifically, the grouping variable is included as a categorical moderator variable, and group differences are determined by estimating the moderation effects. This method is attractive because it allows one to make comparisons across more than two groups for all parameters within a single model and because it is implemented for all commonly used cross-sectional network models. Next to introducing this method, I compare the performance of all above-mentioned methods in a simulation study, a comparison that is currently missing in the literature. Finally, I provide a fully reproducible tutorial on how to estimate group differences in network models with the moderation method using the R-package *mgm* (Haslbeck and Waldorp, 2020).

## Detecting group differences in network models

I first review existing approaches to estimate differences in network model parameters across groups and then introduce the moderation approach.

### Existing approaches

A popular method is the Network Comparison Test (NCT; van Borkulo et al.,, 2017), which is based on a three-step procedure: First, the network model of choice is estimated separately on both groups A, B, and the differences between the parameter estimates in both groups of a parameter serve as the test statistics. Second, for each parameter difference, a sampling distribution under the null hypothesis that there is no difference in the population is created by repeatedly assigning cases randomly to two groups with the same sizes as A, B, and computing the differences in the parameter estimates. This yields a sampling distribution that is centered at zero, since the expected group differences in the permuted groups are equal to zero. Finally, using a specified significance threshold *α*, it is determined whether the test statistic is significantly different from zero. An advantage of this generic method is that it can be applied to essentially any model. This method is implemented in the R-package *NetworkComparisonTest* (van Borkulo et al., 2017).

Epskamp et al., (2020) recently suggested a SEM-based approach to estimating group differences that consists of the following three steps. First, one estimates a model in each separate dataset and removes non-significant partial correlations. Second, a pooled model is estimated in which each parameter is included that was included in at least one of the individual models from the first step. Third, equality constraints are freed in a step-wise fashion until the BIC of the overall model does not improve any further. The model at the end of this procedure is selected as the final model and specifies the estimated group differences. This method is available in the R-package *psychonetrics* (Epskamp, 2020).

Williams et al., (2019) proposed two Bayesian methods to test for differences in parameters across groups. The first method uses a Bayes factor to compare the hypothesis that a given partial correlation is the same in all groups vs. not. The second method computes the posterior of the difference between partial correlations in two groups, and uses a threshold *α* on the posterior to decide whether the difference is reliably different from zero. Both methods are implemented in the R-package *BGGM* (Williams and Mulder, 2019). Recently these methods have been extended to data consisting of continuous, ordinal, and binary variables using a semi-parametric copula model.

Yet another method is the Fused Graphical Lasso (FGL; Danaher et al.,, 2014), which extends the Graphical Lasso (Friedman et al., 2008) with an additional *ℓ*_1_ penalty that includes all group differences. The two penalties are weighted by the regularization parameters *λ*_1_,*λ*_2_ and can be selected using information criteria such as the EBIC, or cross-validation. Thus, group differences are only estimated to be present if they sufficiently increase model fit relative to the additional parameters used (EBIC) or if they increase the out-of-fold prediction error in a cross-validation scheme. The FGL can be used for two or more groups, and is currently implemented for GGMs. The R-package *EstimateGroupNetwork* (Costantini & Epskamp, 2017) implements a grid search of *λ*_1_,*λ*_2_ values using EBIC and cross-validation.

Finally, to test whether partial correlations in GGMs are different across two groups, one can use traditional frequentist methods. For example, one can obtain unbiased estimates of partial correlations in both groups, and transform them with Fisher’s Z-transformation to obtain parameters that are approximately normally distributed (Fisher, 1915). Subsequently, one can use the Z-score of the difference together with a threshold *α* to test the null hypothesis that the difference between the partial correlations is zero. Some of the above methods are also suited to directly test group differences in aggregate parameters. For example, the NCT can be used to test hypotheses about differences in density (average absolute value of interaction parameters) across groups. In this paper, I focus on estimating differences in individual model parameters. However, once group differences of individual parameters are estimated, they can always be aggregated to evaluate hypotheses about aggregates of parameters such as the network density.

### Moderation approach

In this section, I show how to estimate group differences using moderation with categorical moderators. I first illustrate the idea with a simple regression example, and then extend it network models. Consider the simple regression problem:

1 $$ Y = \beta_{YX} X + \varepsilon, $$

involving the variables *X* and *Y*, a parameter *β*_*YX*_ and a Gaussian error term *ε*.

I now introduce an additional categorical variable *G*, which indicates group membership and can take on the values 1, 2, and 3. We are interested in whether the parameter *β*_*YX*_ differs across the three groups, and therefore add the interaction between variables *X* and *G* to the model:

2 $$ Y = \beta_{YX} X + \beta_{Y G_{2} X} \mathbb{I}(G=2) X + \beta_{Y G_{3} X} \mathbb{I}(G=3) X + \varepsilon, $$

where $\mathbb {I}(G=k)$ is the indicator function for the group at hand being group *k*. For example, if G is equal to 3, then the terms $\mathbb {I}(G=1)$ and $\mathbb {I}(G=2)$ are equal to zero, and $\mathbb {I}(G=3)$ is equal to 1.

How do we obtain the main effect of *X* on *Y* in each group from Eq. 2? In group 1, the main effect is equal to *β*_*YX*_; in group 2, it is equal to $\beta _{YX} + \beta _{Y G_{2} X}$; and in group 3, it is equal to $\beta _{YX} + \beta _{Y G_{3} X}$. We see that by adding the grouping variable *G* as an interaction term we obtained the main effect of *X* on *Y* for each group, and therefore also the differences across groups. While taking group 1 as the dummy category in this example emphasizes comparisons between group 1 and the two remaining groups, other comparisons can be computed in a straightforward way from the estimates. For example, the group difference between group 1 and group 2 is $\beta _{YX} + \beta _{Y G_{2} X} - (\beta _{YX} + \beta _{Y G_{3} X}) = \beta _{Y G_{2} X} - \beta _{Y G_{3} X}$. In this example, the grouping variable *G* had three categories (groups), but the grouping variable can also be binary or have more than three categories.

So far, I only considered group differences in main effects. However, groups may also differ in their intercepts. To evaluate such group differences, I include the indicator functions also as main effects in the model. The intercept of each group can then be obtained analogous to the effect of *X* on *Y* in each group as shown above. Instead of the dummy coding used here, one could also use effect coding, which is a statistically equivalent parameterization that allows to directly compare all groups to the grand mean across groups (e.g., Alkharusi, 2012). This might be desirable if the categorical variable is ordinal. However, in that case one could also consider modeling the grouping variable as a continuous variable, which requires less parameters (Haslbeck et al., 2019). Note that the main effects in regression models discussed here relate to pairwise interactions between variables in network models. For example, for the GGM the main effects can be standardized to obtain partial correlations.

We now use the same principle to estimate group differences in network models. This is possible because network models can be estimated with a pseudo-likelihood approach, in which one estimates the conditional distribution of each node, and then combines the estimates obtained from the conditional distributions to the full network model (Besag, 1977; Meinshausen et al., 2006). In the case of the multivariate Gaussian distribution and its implied Gaussian Graphical Model (GGM) with *p* variables, estimating the conditional distribution of a given variable *X*_*i*_ amounts to estimating *p* multiple regression models:

3 $$ X_{i} = \beta_{X_{i} X_{1}} X_{1} + \dots + \beta_{X_{i} X_{p}} X_{p} + \varepsilon, $$

in which each variable $X_{i}, i \in \{1, 2, \dots , p\}$ is predicted by all variables except itself.

Similarly to Eq. 2 in the simple linear regression problem, we can add a grouping variable. Here we choose a binary grouping variable *G*:

4 $$ \begin{array}{@{}rcl@{}} X_{i} &=& \beta_{X_{i} X_{1}} X_{1} + \beta_{X_{i} G_{1} X_{1}} \mathbb{I}(G=1) X_{1} + {\dots} + \beta_{X_{i} X_{p}} X_{p}\\ &&+ \beta_{X_{i} G_{1} X_{p}} \mathbb{I}(G=1) X_{p} + \varepsilon. \end{array} $$

As mentioned above, in the pseudo-likelihood approach each variable is predicted by all other variables in a multiple regression model. This leads to two estimates for each main effect: for example, we obtain the estimate $\beta _{X_{1} X_{4}}$ from the regression on *X*_1_ and the estimate $\beta _{X_{4} X_{1}}$ from the regression on *X*_4_. These two estimates are estimates of the same pairwise interaction between variables *X*_1_ and *X*_4_. These estimates can be standardized to obtain the partial correlation *ρ*_1,4_ in the *p*-variate multivariate Gaussian distribution (Epskamp et al. 2018). To arrive at a single estimate, we aggregate them using the AND-rule (take average) or the OR-rule (take average if both estimates are nonzero, otherwise set to zero). Similarly, we obtain two estimates for the interaction parameters which captures the group differences.

The same procedure can be applied to the Ising model. The only difference is that the nodewise regressions are not linear but logistic regressions (Epskamp et al. 2016). More generally, the procedure can be applied to Mixed Graphical Models (MGMs) (Yang et al. 2014), which generalize both the GGM and the Ising model. In this setting, each variable is a conditional exponential family distribution (e.g., Gaussian, Binomial, Poisson), and one can apply the above procedure by estimating the appropriate regressions in the Generalized Linear Model (GLM) framework. These models are generalized by higher-order (or moderated) MGMs, which are described in detail in Haslbeck and Waldorp (2020). Haslbeck et al., (2019) introduced Moderated Network Models (MNMs) in more detail for the special case of continuous variables. Indeed, the model used here to estimate group differences is a MNM with a categorical moderator. This makes the method very flexible because it can be used within larger MNMs that include several categorical and continuous moderators. This can be an advantage because it potentially allows researchers to answer several research questions by fitting a single model instead of fitting initial network models and evaluating group differences with separate follow-up analyses.

When comparing the moderation approach proposed here with the existing methods discussed in Section “?? ??” in the previous section, the most similar method is the FGL, since it also uses regularization to estimate group differences. The FGL jointly estimates several GGMs using two penalty terms for partial correlations and group differences in partial correlations. The moderation approach proposed here uses a nodewise estimation approach with a single *ℓ*_1_-regularization term that includes both main effects and interactions (which are interactions and moderation effects, respectively, from a graph perspective). While joint estimation is typically preferable to nodewise estimation, the latter selects a different regularization parameter for each variable. This is advantageous when variables differ a lot in how strongly connected they are and how much those connections differ across groups.

### Applicability of different approaches

Table 1 provides an overview of the applicability of the methods so far discussed. The NCT allows one to compare pairs of GGMs, Ising models and MGMs across two groups. The hypothesis test based on Fisher’s z-transformation of partial correlations is only applicable to pairs of GGMs. The FGL allows to compare two or more groups of GGMs. In principle, it could also be implemented for other models, however, currently no such implementation is available. The method based on the Bayes factor compares the hypotheses that two or more partial correlations are the same or not. The method based on thresholding the posterior of differences between partial correlations can compare pairs of GGMs. Using semi-parametric copula models, these two approaches can be extended to ordinal and binary data, however, they cannot handle nominal categorical variables with more than two categories. The SEM-based approach using partial pruning can compare two or more groups and is applicable to the Gaussian and the Ising model. However, detecting group differences between Ising models is only computationally feasible if the number of variables is relatively small (up to around 10).

**Table 1:**
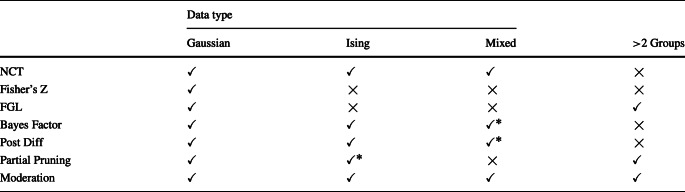
An overview of the adaptability of the applicability of the discussed methods; NCT = Network Comparison Test, Fisher = Hypothesis test based on Fisher’s Z transform; FGL = Fused Graphical Lasso, Post Diff = Thresholding Posterior of Differences

The asterisk for Partial Pruning indicates that the method is only feasible for relatively few variables. The asterisk for the Bayesian methods indicates that these methods cannot handle nominal categorical variables with more than two categories

Finally, the moderation approach allows one to compare MGMs (and therefore also GGMs and Ising models) for more than two groups. Note that, in principle, the NCT, Fisher’s method, and the posterior difference method can be applied repeatedly to compare more than two groups. However, they cannot compare groups in a single application of the method like the FGL, the Bayes factor method and the moderation method.

Another advantage of the moderation approach is that it naturally tests for group differences in all parameters including intercepts. While estimating group differences in intercepts could be added to most methods, the implementation of the moderation approach in the R-package *mgm* and the partial pruning method in the R-package *psychonetrics* (Epskamp, 2020) are currently the only methods that allow to estimate group differences in intercepts.

## Comparing performance of different approaches

In this section I compare the performance of all above discussed approaches in scenarios that are typical for applied research. The goal is both to compare the relative performance of the methods and to provide a general overview of how difficult it is to estimate group differences, which is currently missing in the literature.

### Simulation setup

I focus on estimating differences in GGMs and Ising models across two groups, which represents the most common data analysis problems in applied research and allows us to compare all above discussed approaches.

#### Data generation

I create group differences in the following way: I first create a network model for group 1, and then change a number of parameters in this model to obtain the model for group 2. I then aim to recover those differences with the above discussed methods.

The initial network of group 1 is created such that it resembles the network models encountered in typical psychological applications: I choose network models with *p* = 17 variables, because this is the median number (range 6 − 63) of variables in a recent network re-analysis (Haslbeck and Fried, 2017). In the absence of any general knowledge about network structures in psychology I generate the pairwise dependency structure with a random graph. However, I would not expect the specific type of global graph structure to impact the performance of the moderation method, since it is estimated in a nodewise fashion. Instead, the only graph-characteristic that can influence the performance of the method is the number of neighbors of a given node. I return to this issue when discussing the results of the simulation study. The edge probability in the random graph is set to *P*(edge) = 0.2. That is, on average around 27 of the 136 possible edges are present. In the case of the GGM I assign a draw from a uniform distribution $\mathcal {U}(-0.2, 0.4)$ to each of the edges, which results in values of partial correlations that are commonly observed in empirical data. I set all intercepts to zero. The interaction parameters of the Ising model are roughly three times larger than partial correlations (see Appendix A), and I therefore take draws from $\mathcal {U}(-0.6, 1.2)$ in the case of the Ising model. To ensure that all binary variables have a large enough variance, I set the threshold of each node to the negative sum of edge-weights that are connected to it divided by 1.5. This results in largely negative thresholds, which is what is observed empirically when estimating the Ising model in the {0,1} domain.

In the second step, I create the model of group 2 by randomly picking 20 interaction parameters of the model of group 1, and adding Δ*𝜃* to each of them. This means that 20 out of 136 possible edges (which can be zero or nonzero in group 1) are different across groups, which seems a reasonable scenario for practical applications. Note that this is the important level of sparsity in this simulation, since we are interested in the performance of estimating group differences. In the GGMs, I vary Δ*𝜃* ∈{0.05,0.1,0.2}, in the Ising models I vary Δ*𝜃* ∈{0.15,0.3,0.6}. In the Gaussian case, in rare occasions the partial correlation matrices were not positive definite. In these cases, I sampled repeatedly until the partial correlation matrices of both groups were positive definite.

In order to study performance as a function of sample size, I vary the sample size of *each* group *n* ∈{20,37,68,233,431,795,1467,2708,5000}, which are on a logarithmic scale from 20 to 500. I chose this sequence because it both covers the full range from extremely poor to near-perfect performance, and covers the sample sizes that are typical in psychological applications. I use the above-mentioned methods with a number of different specifications. All methods include tuning parameters that allow one to make the method more liberal/conservative, and which exact tuning parameter is selected in a simulation study is therefore somewhat arbitrary. Here I chose the default values in the respective software packages that are typically used in practice to get a rough overview of the performance of all methods.

#### Estimation

I run the NCT as implemented in the R-package *NetworkComparisonTest* (version 2.2.1; van Borkulo et al.,, 2017) with 1000 permutations (250 for the Ising model to render the simulation feasible; see Appendix C for a demonstration that this reduction does not impact performance), and I use the EBIC for model selection with *γ* = 0.25 and evaluate its performance with significance thresholds *α* = 0.05 and *α* = 0.01. The FGL is estimated with the R-package *EstimateGroupNetwork* (version 0.3.1; Costantini & Epskamp, 2017) using sequential search that first selects the *λ* for the graphical lasso penalty, and then the *λ* for the fused lasso penalty. To select regularization parameters in the FGL, I use tenfold cross-validation and the EBIC with *γ* = 0.25. The Partial Pruning method is run with *α* = 0.05 and *α* = 0.01 using the implementation in the R-package *psychonetrics* (version 0.9; Epskamp, 2020). For the two Bayesian methods, I used the implementations in the *BGGM*-package (version 2.0.3; Williams & Mulder, 2019). For the Bayes factor method, I defined the cut-off value above which a difference is considered reliable as 1. In this method, I set the standard deviation of the Gaussian prior distribution of the group difference to 0.2. The second Bayesian method uses the posterior of the differences between both groups, which I threshold to zero if its 95% credible interval overlaps with zero. Finally, I use the R-package *mgm* version (version 1.2-11; Haslbeck & Waldorp, 2020) for the implementation of the moderation approach. I select the regularization parameter with either 10-fold cross-validation or the EBIC with *γ* = 0.25. I also compare the performance of the algorithm with the AND and the OR-rule. This is especially relevant in the implementation of the *mgm*-package, since it runs a nodewise regression on each node, including the moderator variable. This means that the regression on the moderator variable includes many terms, which renders the AND-rule very conservative. This design is run for 200 iterations. All simulations were performed with R version 4.0.2. The code to reproduce the simulation and all results and figures is available on GitHub: https://github.com/jmbh/NetworkGroupDifferences.

#### Evaluation

I evaluate performance with three different measures. First, sensitivity, which is the probability that a true group difference is recovered. Second, precision, which is the probability that an estimated group difference is a true group difference. These two measures capture how well the methods estimate the presence/absence of group differences. However, they provide a poor measure of how close a given estimate is the true group difference. For example, if the true group difference is 0 and we estimate it to be 0.0001, this error will impact precision a lot, even though the error in the parameter value is tiny, and in practice it is unlikely that one would interpret such a group difference. I therefore also consider the estimation error, which I define as the average absolute value of the difference between true group differences and estimated group differences. For example, if there are two true group differences Δ*𝜃*_1,2_ = 0.1,Δ*𝜃*_6,3_ = 0.3 and the corresponding estimates are equal to 0 and 0.2, then the estimation error is equal to (|0.1 − 0| + |0.3 − 0.2|)/2 = 0.1. I report this estimation error separately for group differences that are present/absent in the true model.

### Simulation results

I report sensitivity (probability of estimating a true group difference), precision (probability that an estimated difference is a true difference) and mean absolute estimation errors ($|{\Delta } \theta - {\Delta } \hat \theta |$) separately for edges that are different or not different across groups. All methods were used in all scenarios, except the partial pruning method was not identified for the sample size *n* = 20. In addition, this method failed to produce estimates in less than 1% of the iterations of the simulation due to issues related with matrix inversion.

#### Results Gaussian graphical model

**Fig. 1:**
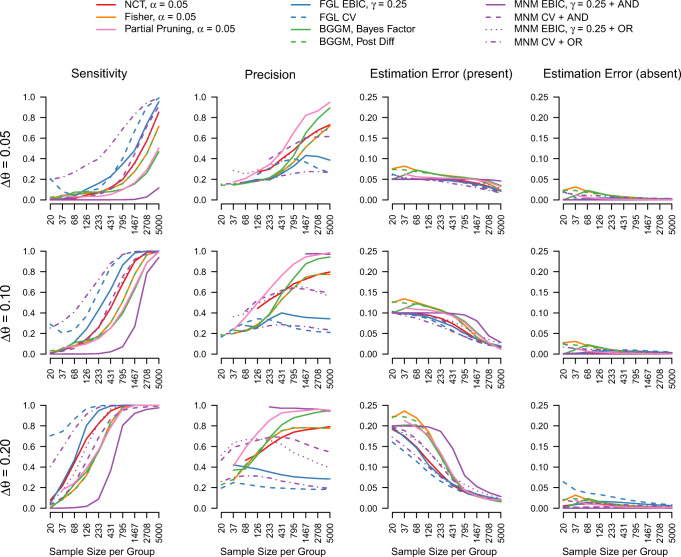
The sensitivity, precision, and estimation errors (separately group differences that are present or absent in the true model) for estimating differences in interaction parameters across groups, for the compared approaches, as a function of Δ*𝜃* and the number of observations *n* in each group. Precision is only displayed for those scenarios in which it was possible to calculate in at least 10% of the iterations. This occurs for scenarios with small *n* and conservative approaches

Figure 1 displays those four performance measures for each method for detecting group differences in GGMs, as a function of the size of the group difference Δ*𝜃* and sample size per group *n*.

I first focus on the estimation of true group differences in columns one and three of Fig. 1. Sensitivity increases for all methods, as expected, however at different rates. The larger the true group difference Δ*𝜃* the quicker sensitivity increases as a function of *n*, which is what one would expect. Within each Δ*𝜃*-variation, the estimation methods differ in how quickly sensitivity increases with *n*. That is, the methods differ in how liberal/conservative they are. What stands out is that the MNM with cross-validation (CV) and the OR-rule is most the liberal, and that the MNM with EBIC and the AND-rule is the most conservative. Also, the FGL approaches seem to be more liberal than the NCT, which in turn is more liberal than the Fisher’s method. The latter is followed by the Bayesian methods which have a similar sensitivity as the partial pruning method. While sensitivity tells us how well a method is performing in determining whether a group difference is present or absent, it does not tell us how close the estimate is to the true difference.

The estimation error for true group differences in the third column of Fig. 1 shows how close the estimates are to the true group differences. We see that the methods without regularization have a relatively large estimation error when *n* is small. The regularized methods, on the other hand, seem to never have an estimation error that is larger than the true group difference Δ*𝜃*. This is because methods without regularization can show errors both due to over- and underestimation, while the methods with regularization tend to show only error due to underestimation. Therefore the largest error they can show is Δ*𝜃*. While the estimation error provides additional information to sensitivity, the two measures are of course strongly related: The higher sensitivity, the lower the estimation error for present group differences.

Next, we consider how well absent group differences are estimated. We only display precision if differences were estimated to be present in at least 90% of the iterations. This is to avoid precision estimates that are based only on a few iterations. I also do not show the performance of the Partial Pruning method for *n* = 20, since the underlying model was not always identified in this scenario. We see that precision is slightly higher for larger group differences, which makes sense because the presence of true group differences is easier to estimate. We also see that the methods roughly stack up in the reverse order compared to sensitivity, as one would expect from methods that offer different trade-offs between sensitivity and precision. The MNM methods with AND-rule are very conservative, because they also include a regression on the moderator variable, which includes a very large number of terms and therefore the regularization sets most terms to zero.

All methods seem to converge to a sensitivity of 1, however, not all methods seem to converge to a precision of 1. This is at least the case for the FGL approaches and some of the MNM approaches. However, precision does not tell us how large the estimates of those false positive group differences are. I therefore display the estimation errors for absent group differences in the fourth column of Fig. 1. For low *n* the unregularized methods show high estimation errors, which tend to 0 as *n* increases. The regularized methods show very low estimation errors for all *n*. This shows that while these methods incorrectly estimate some group difference to be present, these incorrect estimates are extremely small. We also see that this estimation error does not differ across Δ*𝜃* which makes sense, because the absent edges do not vary across these scenarios.

**Fig. 2:**
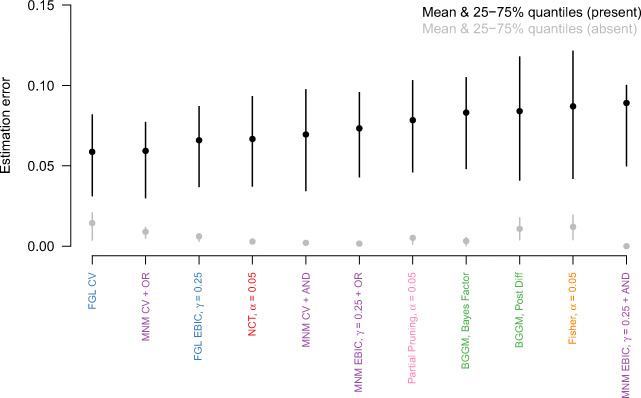
The mean estimation errors aggregated over sample sizes and Δ*𝜃*, ordered from smallest to largest estimation error for present group differences. The *vertical lines* indicate the 25% and 75% quantiles of the estimation errors

While all methods are similar in that they offer different trade-offs between correctly estimating present vs. absent group differences and in that they are performing very well for high *n*, there do exist differences in absolute performance. To get a better picture of these, I display the mean errors across sample sizes and Δ*𝜃* in Fig. 2.

The figure orders the methods from smallest to largest estimation error for present group differences (black points). When comparing these errors to the estimation errors for absent group differences (grey points), we see that for many methods there is a trade-off between the two estimation errors. This is especially pronounced for the first six methods: For example, the FGL with CV has low estimation errors for present group differences, but high estimation errors for absent ones. For the MNM method using CV and the AND-rule the opposite is true. However, some methods seem to perform worse overall, such as Fisher’s exact test, the Bayesian method based on thresholding the posterior of group differences, and the partial pruning method.

#### Results Ising model

**Fig. 3:**
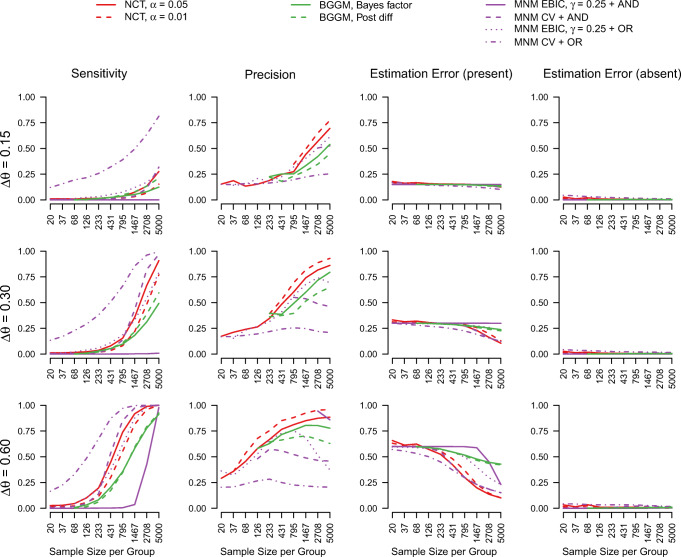
The sensitivity, precision, and estimation errors (separately group differences that are present or absent in the true model) for estimating differences in interaction parameters across groups, for the compared approaches, as a function of Δ*𝜃* and the number of observations *n* in each group. Precision is only displayed for those scenarios in which it was possible to calculate in at 10% of the iterations. This occurs for scenarios with small *n* and/or conservative approaches

Figure 3 displays sensitivity, precision, and estimation errors for each method for detecting group differences for the Ising model, as a function of the size of the group difference Δ*𝜃* and sample size *n*.

We again focus first on the estimation of present group differences. Similarly to the GGM, sensitivity increases fast with *n* with larger true group differences Δ*𝜃*. For the estimation of group differences in the Ising model only the NCT and the MNM methods are applicable. Similarly to the GGM results above, the most liberal method is the MNM with CV and the OR-rule, and the most conservative method is the MNM with EBIC and the AND-rule, for the same reasons as in the GGM case. The remaining methods show comparable sensitivity. The estimation error for present group differences never exceeds the true group difference, which is because both the NCT and MNM methods use regularization which biases estimates of group differences towards zero.

Next, we consider the estimation of absent group differences. As expected, the precision of the methods stacks up in reverse order compared to sensitivity. Again, similarly to the GGM, some MNM methods do not seem to approach 1 as *n* increases, but the estimation errors in the fourth column show that these errors are very small across all *n*.

**Fig. 4:**
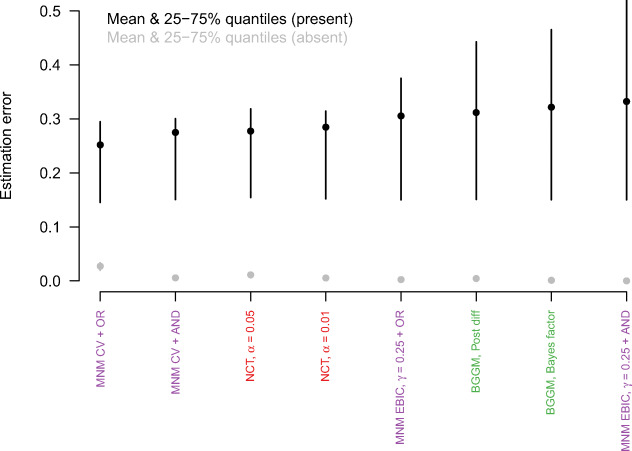
The mean estimation errors aggregated over sample sizes and Δ*𝜃*, ordered from smallest to largest estimation error for present group differences. The *vertical lines* indicate the 25% and 75% quantiles of the estimation errors

Again, we inspect the estimation errors across sample size and Δ*𝜃* to get a better picture of the overall performance (see Fig. 4). Similarly to the Gaussian case, we observe a trade-off between low estimation error in present vs. absent group differences. For example, the MNM method with CV and the OR-rule has the lowest error for present edges and the highest error for absent edges, while the reverse is true for the MNM method with the EBIC and the AND-rule.

#### Summary of results

To summarize, we saw that no method clearly outperforms any other method, but that they offer different trade-offs between correctly estimating present and absent group differences. When considering estimation errors, no method performs much worse than any other method, and the method that does worse in estimating the present differences, does best in estimating the absent ones (and vice versa). If one is interested in estimating whether a group difference is present or not, one needs to consider sensitivity and precision. In that case, the considered methods provide different trade-offs between the two error measures, however, a few method show very low precision (FGL, MNM methods with OR-rule).

The results above may suggest that these trade-offs are mostly a function of the type of method. However, there are also considerable differences within a given method, depending on which tuning parameters are chosen. We see this especially for the FGL and MNM methods. In Appendix B, I report additional simulation results for other choices of tuning parameters of the remaining methods (e.g., *α* = 0.01 vs. *α* = 0.05). This shows that the trade-off between sensitivity/precision is both a function of the type of method and the particular choice of tuning parameters. Some methods do not seem to converge in precision; however, the estimation errors show that the errors that lead to low precision are very small. In general, we saw that a considerable number of observations is necessary in each group in order to recover small group differences. If group differences are large, however, they can be picked up even with small sample sizes per group around *n* = 100 to 200. Finally, assuming that the relative scaling of GGM and Ising model parameters I chose is reasonable, it is much harder to detect group differences in the Ising model.

In this simulation, I kept the sparsity of the base-graph constant at *P*(edge) = 0.2. If the estimation of group differences is performed separately for each parameter, such as in Fisher’s exact test, Partial pruning, and the Bayesian methods, one would expect that the performance is largely unaffected. However, if estimating a given parameter to be present depends on whether other parameters are present in the true model, such as in methods using regularization, one would expect that the performance is affected. Specifically, the estimation methods using regularization will become more liberal if sparsity becomes smaller. This is because the selected regularization parameters will be small, which results in less parameters being shrunk to zero. These predictions are verified in Appendix D.

The runtime of all methods is relatively fast for the GGM, with the exception of the FGL with cross-validation. For the Ising model, the runtime of the NCT and the Bayesian methods increases substantially with sample size, while the runtime of the moderation approach remains relatively low (for details see Appendix E).

## Tutorial on estimating group differences in mixed data with the moderation approach

In this section, I show how to use the R-package *mgm* (Haslbeck & Waldorp, 2020) to estimate differences across three groups in mixed data using the moderation approach. To be able to share the data and therefore to make the tutorial fully reproducible for the reader, I use simulated data. The data consists of four continuous variables, two (nominal) categorical variables with two and three categories, and a categorical moderator with three categories, which serves as the grouping variable. Appendix F describes how I generated the dependencies between these variables.

The data are automatically loaded with the *mgm*-package and can be accessed in the object dataGD. The dataset contains 3000 observations, 1000 in each of the three groups of the grouping variable in column 7:


library(mgm)> dim(dataGD)[1] 3000 7> head(dataGD) x1 x2 x3 x4 x5 x6 x7[1,] 0.214 0.157 0 -0.624 1 0.105 1[2,] 0.480 0.743 1 -0.273 1 0.428 1[3,] 0.088 -0.129 1 1.326 0 -0.963 1[4,] 0.444 -0.487 1 1.670 0 0.641 1[5,] -0.363 -0.080 1 0.306 2 0.376 1[6,] 0.123 -0.940 0 -0.605 0 1.000 1

In order to detect group differences in the Mixed Graphical Model describing the relationships between variables $X_{1}, X_{2}, \dots , X_{6}$, we fit a moderated MGM with the grouping variable *X*_7_ being specified as a categorical moderator:


mgm_obj <- mgm(data = dataGD, type = c("g", "g", "c", "g", "c", "g", "c"), level = c(1, 1, 2, 1, 3, 1, 3), moderators = 7, lambdaSel = "EBIC", lambdaGam = 0.25, ruleReg = "AND")

The argument type indicates the type of variable (“g” for continuous Gaussian, and “c” for categorical) and level indicates the number of categories of each variable, which is set to 1 by convention default for continuous variables. The moderators argument specifies that the variable in the 7^*th*^ column is included as a moderator. Since we specified via the type argument that this variable is categorical, it will be treated as a categorical moderator. The remaining arguments specify that the regularization parameters in the *ℓ*_1_-regularized nodewise regression algorithm used by *mgm* are selected with the EBIC with a hyperparameter of *γ* = 0.25 and that estimates are combined across nodewise regressions using the AND-rule.

In order to inspect the MGMs in the three groups, we need to condition the moderated MGM on the values of the three groups. This can be done with the function condition(), which takes the moderated MGM object and a list specifying on which values of which variables the model should be conditioned on. Here we only have a single moderator variable (*X*_7_) and we condition on each of its values {1,2,3} which represent the three groups, and save the three conditional pairwise MGMs in the list object l_mgm_cond:


l_mgm_cond <- list()for(g in 1:3) l_mgm_cond[[g]] <- condition(mgm_obj, values = list("7" = g))

We can now inspect the pairwise MGM in each group similar to when fitting a standard pairwise MGM (for details see Haslbeck & Waldorp, 2020). Here I choose to visualize the strength of dependencies in the three MGMs in a network using the *qgraph* package (Epskamp et al., 2012). We provide the three *mgm* objects as an input and set the maximum argument in qgraph() for each visualization to the maximum parameter across all groups to ensure that the visualizations are comparable.


library(qgraph)v_max <- rep(NA, 3)for(g in 1:3) v_max[g] <- max(l_mgm_cond[[g]]\textit{pairwise}wadj)par(mfrow=c(1, 3))for(g in 1:3) qgraph(input=l_mgm_cond[[g]]\textit{pairwise}wadj, edge.color = l_mgm_cond[[g]] \textit{pairwise}edgecolor, layout = "circle", mar=c(2, 3, 5, 3), maximum = max(v_max), vsize = 16, esize = 23, edge.labels = TRUE, edge.label.cex = 3) mtext(text= paste0("Group ", g), line= 2.5)

**Fig. 5:**
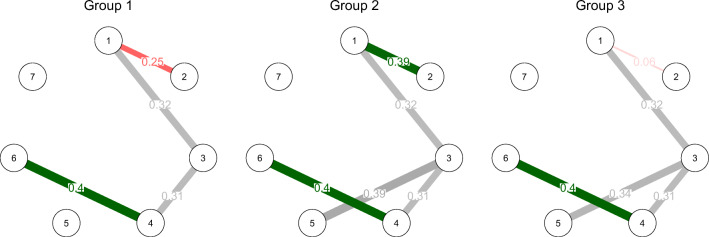
The conditional MGMs in the three groups obtained by conditioning the moderated MGM on the values of the grouping variables. *Green edges* indicate positive linear relationships, *red edges* indicate negative linear relationships, *grey edges* indicate relationships involving categorical variables, for which no sign can be defined. The width of edges is proportional to the strength of the dependency

The resulting network visualization is shown in Fig. 5. The edges represent conditional dependence relationships and their width is proportional to their strength. The green (red) edges indicate positive (negative) linear relationships. The grey edges indicate relationships involving categorical variables, for which no sign is defined (for details see Haslbeck & Waldorp, 2020). We see that there are conditional dependencies of equal strength between variables *X*_1_ − *X*_3_, *X*_3_ − *X*_4_ and *X*_4_ − *X*_6_ in all three groups. However, the linear dependency between *X*_1_ − *X*_2_ differs across groups: it is negative in group 1, positive in group 2 and almost absent in group 3. In addition, there is no dependency between *X*_3_ − *X*_5_ in group 1, but there is a dependency in groups 2 and 3. Note that the equal strength in dependencies between those two categorical variables in groups 2 and 3 does not mean that the exact nature of these dependencies is the same, because the edge weight is an aggregate over a larger set of parameters specifying this categorical-categorical interaction. In situations with many (small) group differences a direct visualization of group differences may be more clear. I provide such a visualization for the present tutorial example in Appendix G.

Alternatively to conditioning the moderated MGM one can also inspect the parameters of the moderated MGM directly. As with pairwise MGMs, this is possible to inspect the (non-aggregated) parameter estimates of these interactions with the function showInteraction(). In this case, it is important to keep the interpretation of pairwise and moderation effects in mind. If a given pairwise dependency is moderated (i.e., differs across groups), then the pairwise interaction indicates the pairwise interaction of the reference group. The smallest value of the categorical moderator variable is used as the reference category by default.

## Discussion

In this paper, I introduced a new way to estimate group differences in statistical network models by treating the grouping variable as a moderator. This method is attractive because it allows to compare networks across more than two groups, it is easy to implement for many models, it can be used within larger MNMs, and is very fast. In addition, I presented a simulation study that evaluated the performance of the moderation method and existing methods. Finally, I provided a fully reproducible tutorial on how to compare an MGM across three groups.

The simulation results showed that different methods provide a different trade-off between correctly estimating present and absent group differences. When considering sensitivity and precision, we saw that the considered methods showed different trade-offs between the two measures: the higher a method scored on sensitivity, the lower it tended to score precision. However, some methods (FTL, MNM with OR-rule) show very low precision, which may be unacceptable in some applications. When considering estimation error, the methods again trade-off between low errors for present vs. absent group differences. Considering the estimation errors also showed that methods with low precision can still have a very low estimation error, because a group difference is counted as incorrectly estimated to be present even if the estimated group differences is extremely small. While all methods trade-off estimation error on present vs. absent group differences, some methods performed overall worse, such as Fisher’s test and the Bayesian method based on thresholding the posterior of group differences. The same qualitative results held true for the Ising model, except that no method showed overall poor performance in this setting.

Now, which method should researchers use in practice? I have shown that some methods have a somewhat lower overall performance in the Gaussian setting, which should consequently be avoided if the applied setting resembles the one of the simulation in this paper. The remaining methods offer different trade-offs between correctly estimating present vs. absent group differences. Which trade-off is more appropriate has to be decided based on the research goals of the study at hand. For example, in an exploratory study, it might be acceptable to trade some precision for more sensitivity to detect group differences. In addition, I showed in the simulations in the appendix that the regularized methods are sensitive to the sparsity of the networks: if a network is highly connected, the regularization-based estimators become more liberal. The running time was low in the Gaussian case for all methods except the FGL method with cross-validation. For the Ising model, the NCT and the Bayesian methods took considerably longer to run than the MNM methods for large *n*. However, if one analyzes only a few data sets the running time is likely to be a minor consideration.

There are several limitations about the reported simulation study that require discussion. First, the group sizes are equal, which is typically not the case in practice. I assumed that the data is Gaussian, however in practice data are often skewed. Similarly, in the Ising model I generated the true models such that all variables have reasonably large variance, which is often not the case in empirical data. The performance reported in the simulation study should therefore be interpreted as the best-case scenario. Also, I only considered comparisons between pairs of groups, in order to be able to compare most of the currently used methods. However, I expect that the performance is similar for three or more groups, as long as the sample size in each group remains constant. The effect sizes for group differences (or moderation effects) are typically small in observational behavioral datasets (e.g., Sherman & Pashler, 2019; Chaplin, 1997; McClelland & Judd, 1993). The true effect sizes are therefore likely to be close to Δ*𝜃* = 0.05 or Δ*𝜃* = 0.15 for Gaussian and Ising models, respectively. This suggests that one needs a relatively large sample size to estimate group differences reliably in observational data. Throughout the simulation study I focused on the comparison of interaction parameters. However, one could also be interested in group differences in the intercepts. Such differences are likely easier to estimate since they involve lower-order interactions. While group differences can in principle estimated for most of the methods considered in this paper, implementations are currently only available for the MNM methods and the partial pruning method. In the implementation of the moderation approach used in this paper, group differences were estimated regularized GLMs with interaction terms. This means that one does not have a theoretically guaranteed false positive rate *α* or confidence intervals. However, one could also perform hypothesis tests on the interaction parameters, or one could use the desparsified LASSO (van de Geer et al., 2014) to obtain unbiased sampling distributions to construct confidence intervals.

An avenue for future research would be to find a more elegant way to combine nodewise regressions in the moderation approach. Currently, the AND-rule is extremely conservative, because it includes the regression on the grouping variable, which has a huge number of parameters compared to the other two regressions. On the other hand, the OR-rule is very liberal, since one nonzero estimate out of three is sufficient to estimate a group difference to be present. A better aggregation rule could be to use an AND-rule only on the regressions that do not predict the grouping variable. Another interesting question is how the different methods compare on graph structures that are different from random networks. While methods such as Fisher’s test, partial pruning and the Bayesian methods are likely to be affected relatively little, there could be differences between the regularized methods using nodewise (NCT, MNM) and global (FGL) estimation.

To summarize, I introduced a new method to detect group differences between network models based on moderation analysis, which goes beyond existing methods in that it allows one to compare all parameters in networks across several groups and grouping variables, and is available for the GGMs, the Ising models, and MGMs. In addition, I provided the first comprehensive simulation study comparing the performance of existing methods to estimate group differences in network models. I hope that these results help applied researchers to choose the best method in a given situation and to better plan studies that involve investigating group differences.

## Figures and Tables

**Fig. 6:**
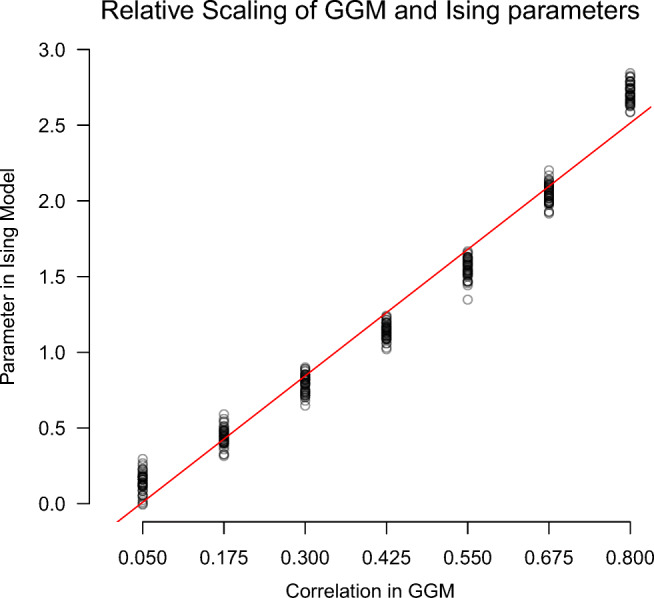
The relative scaling of correlations in GGMs and parameters in the Ising model. The red line indicates the best fitting regression line

**Fig. 7:**
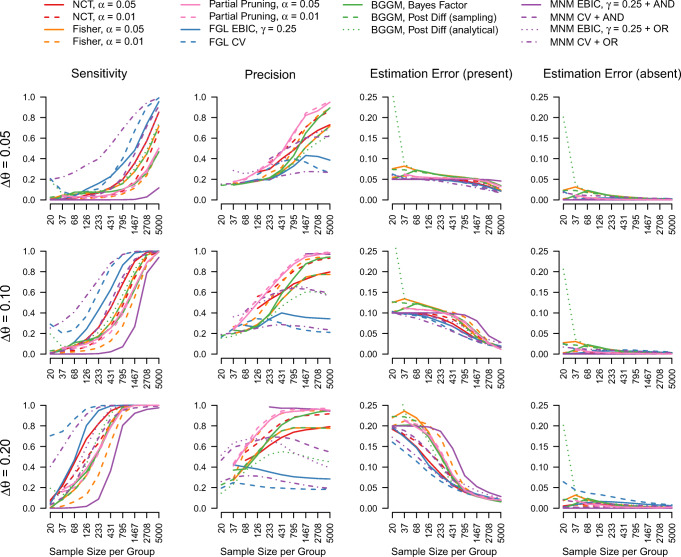
The sensitivity, precision, and estimation errors (separately group differences that are present or absent in the true model) for estimating differences in interaction parameters across groups, for the compared approaches, as a function of Δ*𝜃* and the number of observations *n* in each group

**Fig. 8:**
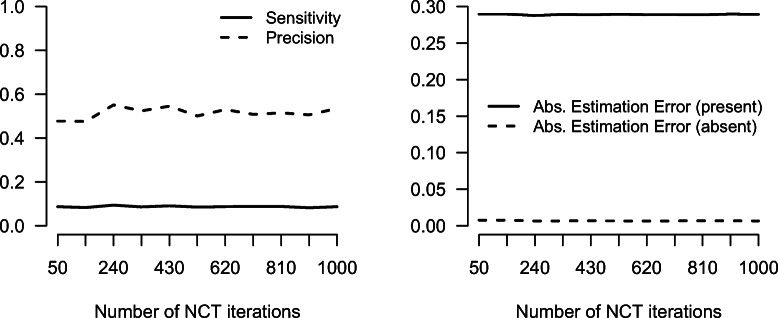
The sensitivity, precision, and estimation errors (separately group differences that are present or absent in the true model) for *n* = 500 (per group) and Δ*𝜃* = 0.3. All other model specifications are as described in Section “Simulation setup”

**Fig. 9:**
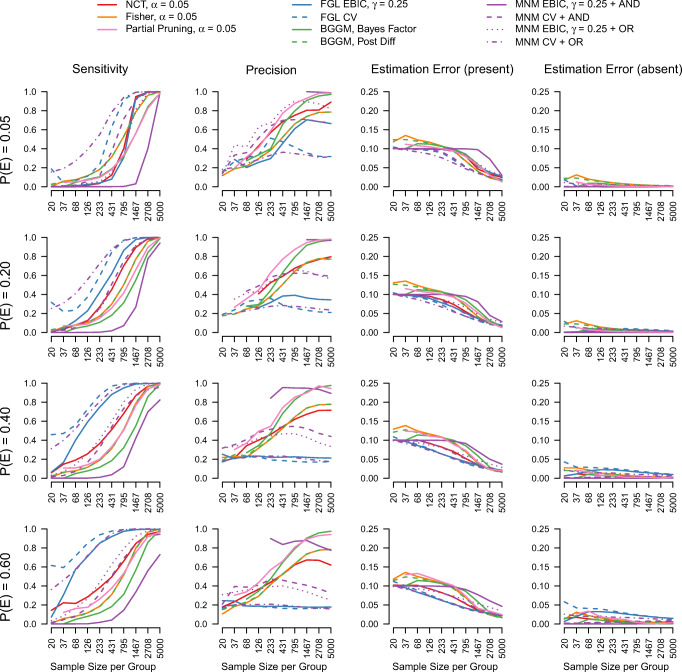
The sensitivity, precision, and estimation errors of all methods for different levels of sparsity of the base graph and different sample sizes per group, for a fixed Δ*𝜃* = 0.1

**Fig. 10:**
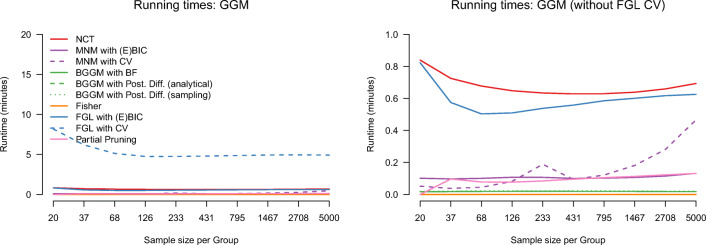
The runtime (minutes) of all methods as a function of sample size per group for the GGM. Left panel: all methods; right panel: all methods except the FGL with CV

**Fig. 11:**
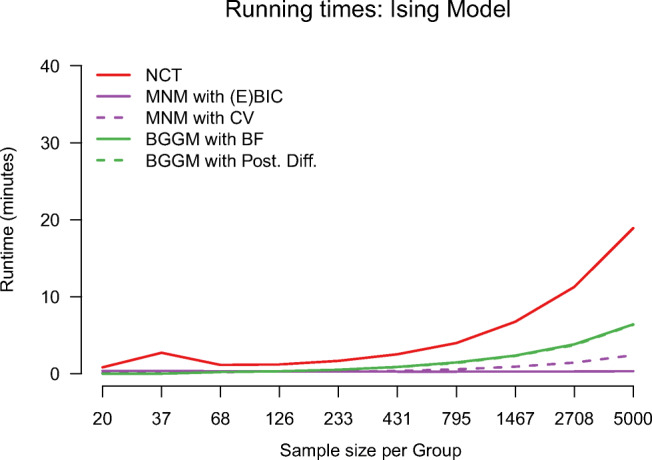
The runtime (minutes) of all methods as a function of sample size per group for the GGM

**Fig. 12:**
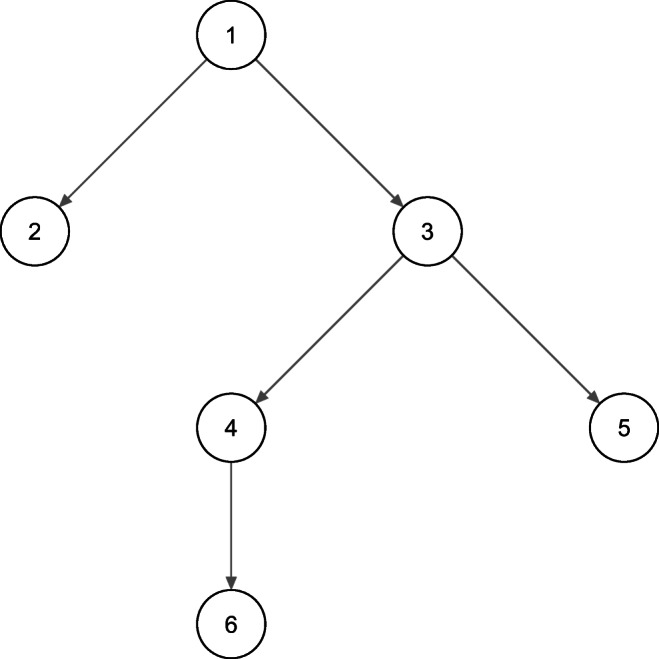
The definition of the mixed DAG used to generate the data for group 1

**Fig. 13:**
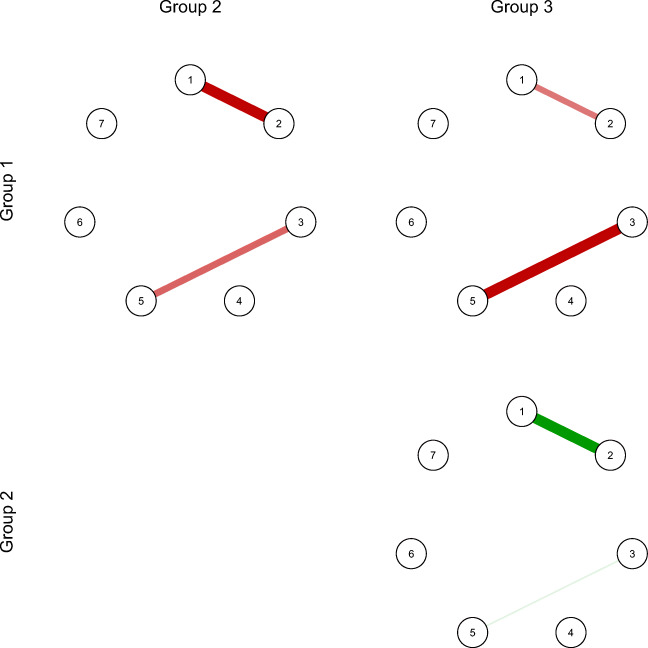
An alternative visualization of group differences in edge-weights: Networks of the differences in edge-weights between group 1 - group 2, group 2 - group 3, and group 2 - group 3 (clockwise)

## Appendix: Appendix 1: Relative scaling of parameters in GGM and Ising model

In order to make the simulation results for the GGM and the Ising model somewhat comparable, I conducted the following small simulation to gauge the relative size of parameters in the two models. To do so, I simulate from multivariate Gaussian distributions with five dimensions that have a single nonzero correlation, and I vary the value of this correlation between 0.05 and 0.80. Then I binarize the data at the median and obtain unbiased estimates for the Ising model in the {0, 1} domain. The simulation is repeated 50 times. Figure 6 displays the relationship between correlations in GGMs and parameters in the Ising model.

We see that the relationship can be reasonably well approximated with a linear function. The parameters of the best fitting regression line are *β*_0_ = − 0.16 and *β*_1_ = 3.3 (red line). I therefore choose the parameters and group differences in the Ising model to be three times larger than in the GGM.

## Appendix: Appendix 2: Additional GGM simulation results

In Fig. 7 we present the results of additional variations of the algorithms shown in Fig. 1. We show the performance of the NCT, the Fisher’s method, and the Partial Pruning method also for *α* = 0.01 instead of *α* = 0.05. We also show the analytic solution of the Bayesian posterior difference method. The key observation is that the sensitivity/precision trade-off differs also considerably within methods, depending on the particular choice of hyperparameters.

## Appendix: Appendix 3: Performance of NCT as function of number of iterations

In the simulation on estimating group differences in the Ising model, we reduced the number of iterations of the NCT from the default of 1000 in the *NetworkComparisonTest* package (version 2.2.1; van Borkulo et al.,, 2017) to 250 in order to render the simulation feasible. This reduction might negatively impact the performance of the NCT, which would mean that we underestimate the performance of NCT as it is commonly used for estimating group differences in the Ising model.

I therefore performed a simulation study to determine the effect of the number of iterations on the NCT performance. To render the simulation feasible, I pick a single condition (*n* = 500,Δ*𝜃* = 0.3) from the simulation study reported in the main text, and vary the number of iterations in eleven steps from 50 to 1000. Figure 8 displays the four performance measures, averaged over 50 simulation iterations, separately for the four performance measures as a function of the number of iterations in NCT. We see that the performance of the NCT is unaffected by the number of iterations in the considered range. This means that the reduction of the iterations from 1000 to 250 in the main simulation does not lead to an underestimation of the NCT performance in detecting group differences in the Ising model.

## Appendix: Appendix 4: Performance of methods with different sparsity

In the simulation study reported in the main text, we fixed the sparsity of the base network of group 1 to *P*(edge) = 0.2. Here we discuss how the sparsity of the model influences the different methods for estimating group differences. We would expect that methods that determine group differences locally are not affected by sparsity. For example, Fisher’s exact test considers partial correlations separately, and hence it is not directly influenced by how many other partial correlations are nonzero. Similarly, the Bayesian methods using the Bayes factor and the thresholding of the posteriors of the differences, and the partial pruning method determine group differences locally.

In contrast, the FGL- and the MNM-based methods are determining group differences by estimating the complete model. Here sparsity matters for the following reason: The lower sparsity, the more parameters are nonzero, which means that those methods will select a smaller regularization parameter. This results in higher sensitivity for pairwise interactions (see Appendix F in Haslbeck et al.,, 2019 for an illustration of the same effect). The same holds for moderation effects (i.e., group differences), which are parameters in the same model. Of course, this higher sensitivity comes at the cost of lower precision. The NCT is an in-between case. It tests group differences locally, but it uses regularization. If sparsity is high and most parameters are zero, the regularization parameters are high, and many parameters get set to zero. This reduces sensitivity, because group differences of parameters close to zero are not being detected. One would therefore expect that NCT is more affected by varying sparsity than the local methods, but less than the FGL- and MNM-based methods. In addition, one would expect that the biggest differences are for small *n*.

To evaluate these theoretical expectations empirically, I ran another simulation study for the Gaussian case in which I held Δ*𝜃* constant at 0.1 and varied *P*(edge) ∈{0.05, 0.40, 0.60}. In all other aspects the simulation is the same as the one reported in the main text, except that the simulation was run for 100 iterations instead of 200. I then combined the results with the ones from the main text where Δ*𝜃* = 0.1 in Fig. 9.

As expected from theory, the performance of the local methods is largely unchanged by the variation of sparsity, while the global methods become more liberal with increasing sparsity. Note that we could also vary another type of sparsity, which is the sparsity of group differences. Since these are referring to moderation effects instead of pairwise interactions in the overall model, varying this type of sparsity would have the same effect as the sparsity of the base-graph explored here.

## Appendix: Appendix 5: Runtime of different methods as function of sample size

Figure 10 displays the runtime of all methods in minutes as a function of sample size per group for the GGM.

We see that all methods run fairly quickly below one minute. The only exception is the FGL with cross-validation. The reason is that searching the grid over two regularization parameters is computational very expensive, even using the sequential search used in our simulation.

Figure 11 displays the runtime of all methods in minutes as a function of sample size per group for the Ising model. We see that the runtime of the NCT and the Bayesian methods increase substantially with sample size. In contrast, the runtime of the moderated regression-based approach grows only slowly with sample size.

## Appendix: Appendix 6: Generation of data used in tutorial

This appendix describes how I generated the data used in the tutorial. To generate the data in group 1, we used the DAG shown in Fig. 12 for mixed variables.

To generate data for group 2, I changed the linear effect of *X*_1_ on *X*_2_ from − 0.5 to 0.5, and I changed the probability table for the relationship between *X*_3_ and *X*_5_ to

In order to generate data for group 3, I adapted the model of group 1 by removing the linear effect of *X*_1_ on *X*_2_ and by changing the probability table for the relationship between *X*_3_ and *X*_5_ to

Thus in the population model there are two dependencies that differ across groups. The dependency between the continuous variables *X*_1_ and *X*_2_ is negative in group 1, positive in group 2 and absent in group 3. The dependency between the categorical variables *X*_3_ and *X*_5_ is present in different forms in groups 2 and 3, and it is absent in group 1 (all six cells have the same probability of $\frac {1}{6} \approx 0.166$).

I sampled 1000 observations from each DAG and combined them into the dataset used in the tutorial. The code to generate this dataset with the model described above is available on GitHub: https://github.com/jmbh/NetworkGroupDifferences.

## Appendix: Appendix 7: Visualization of group differences in networks

In the tutorial I displayed group differences by showing the parameters in each group next to each other in a network visualization. However, when there are many and many small differences, it might be better to instead display the differences in parameters across pairs of groups. We provide such a visualization in Fig. 13.
